# Study on the Inhibitory Mechanisms of Native Cellulose, Microcrystalline Cellulose, and Soluble Cellodextrin on α-Amylase and Amyloglucosidase

**DOI:** 10.3390/foods15010051

**Published:** 2025-12-24

**Authors:** Yanli Zhu, Lin Su, Shanshan Liu, Liping Lu, Li Song, Huimin Ma, Mingyue Zhang, Dandan Gao

**Affiliations:** 1 Key Laboratory of Biotechnology and Bioengineering of State Ethnic Affairs Commission, Northwest Minzu University, Lanzhou 730030, China; zyanli0129@163.com (Y.Z.); 18203940701@163.com (L.S.); 2 School of Life Sciences and Engineering, Northwest Minzu University, Lanzhou 730030, China; liushshy@163.com (S.L.); llp901030@126.com (L.L.); 19859703374@163.com (H.M.);; 3 Gansu Hualing Dairy Company Ltd., Hezuo 747000, China; 18919859183@163.com

**Keywords:** cellulose, amylase, inhibition mechanism

## Abstract

The inhibitory effects of natural cellulose (NC), microcrystalline cellulose (MC), and soluble fiber dextrin (SC) on amylase activity have been established; however, the underlying mechanisms remain poorly understood. This study employed fluorescence spectroscopy and fluorescence thermodynamics to investigate the quenching parameters, thermodynamic properties, and quenching mechanisms of cellulose interactions with α-amylase and amyloglucosidase. Structural alterations in both enzymes were examined using synchronous fluorescence and UV–visible absorption spectroscopy. The results indicated that NC, MC, and SC primarily induced static quenching of fluorophores in α-amylase and amyloglucosidase. When addition of SC reached 3%, SC reduced the fluorescence intensity of tyrosine and tryptophan residues in α-amylase by 70.9% and 86.8%, and in amyloglucosidase by 43.7% and 46.5%, respectively. Increasing SC levels also decreased hydrophobicity around tyrosine and tryptophan in α-amylase. These findings provide insights into designing cellulose-based amylase inhibitors through structural modulation for developing low-glycemic index (GI) foods.

## 1. Introduction

Starch is one of the most abundant natural carbohydrates, providing 40–80% of the body’s energy requirements and playing a vital role in regulating blood glucose levels [1]. Rapid starch digestion causes sharp increases in postprandial blood glucose and insulin levels, contributing to chronic hyperglycemia [2,3,4]. At the same time, the prevalence of other digestion-related conditions—such as hypertension, obesity, atherosclerosis, colon cancer, and cardiovascular disease—has also increased [5,6,7,8]. Therefore, controlling the rate of starch digestion and absorption and carefully regulating starch intake is essential, especially for individuals with diabetes, as it offers a safer and more effective strategy for managing glucose levels.

α-Amylase and glucoamylase serve as key enzymes in the intestinal metabolism of starch [9,10]. Inhibiting the activity of these enzymes can slow starch breakdown, reduce glucose absorption rates, and help manage postprandial blood glucose levels. Although synthetic amylase inhibitors—such as acarbose, miglitol, and voglibose—are currently used to regulate blood sugar, their prolonged use is often associated with gastrointestinal side effects, including bloating, diarrhea, and abdominal discomfort [11,12]. Therefore, there is growing interest in natural amylase inhibitors due to their potential for improved safety and efficacy.

Cellulose, one of the most abundant dietary fibers (DFs) in nature, has demonstrated the potential to delay starch digestion [13,14]. It is composed of long-chain macromolecules made up of D-glucopyranose units linked by β-1,4 glycosidic bonds and lacks any branching. Each glucose unit contains three hydroxyl groups capable of forming hydrogen bonds, especially between the hydroxyl groups at the C3 position and the oxygen atoms of adjacent glucose rings. These hydrogen bonds contribute to the linearity and rigidity of the cellulose structure [15]. The extensive intra- and intermolecular hydrogen bonding compact cellulose chains into tightly packed, crystalline regions that are highly resistant to chemical penetration. Although cellulose contains numerous hydroxyl groups that readily absorb water and promote swelling, these groups alone lack sufficient hydrolysis power to overcome the strong intermolecular forces within the cellulose structure. As a result, cellulose can swell in water but does not dissolve [16]. Along with the tightly packed crystalline regions, cellulose also contains amorphous, non-crystalline areas. In these regions, water molecules can form hydrogen bonds with a limited number of free hydroxyl groups and create water bridges between cellulose chains, leading to swelling [17]. Qi et al. [18] reported that rice bran insoluble dietary fiber (IDF) lowered postprandial blood glucose levels by binding glucose and inhibiting α-amylase activity. Similarly, Dhital et al. [19] demonstrated that cellulose reduced the rate of starch hydrolysis through nonspecific adsorption of α-amylase, with the extent of inhibition depending on the cellulose concentration. However, other studies have observed minimal inhibitory effects of nanofibrillated cellulose on α-amylase and α-glucosidase at 1% concentration, though a slight reduction in lipase activity was observed [20]. However, detailed analysis of how cellulose with distinct structural characteristics affects amylase activity has not been conducted in existing studies. The crystalline structure, solubility, and particle morphology of cellulose may alter its direct interaction with enzymes. As previously mentioned, the crystallinity of cellulose is influenced by its source and preparation method [21,22,23]. Modifying the crystalline structure of cellulose can enhance its water-holding capacity and swelling ability. Nevertheless, it remains unclear how changes in crystallinity regulate cellulose’s inhibitory effect on amylase activity, as well as how the binding affinity between cellulose and enzyme active sites is affected. Changing the crystalline structure can enhance the water-holding and swelling capacities of cellulose. In our previous study, three types of cellulose with distinct crystalline structures were prepared using ultrasound-assisted acid hydrolysis. Natural cellulose (NC) displayed a long-chain, entangled structure with significantly higher swelling capacity, water-binding ability, and apparent viscosity compared to microcrystalline cellulose (MC) and soluble fiber dextrin (SC). MC consisted of aggregated small fiber particles, while SC had short-rod shapes with a completely amorphous structure. The relative crystallinities of NC and MC were 35.5% and 49.5%, respectively. SC was composed of an amorphous structure as confirmed by X-ray diffraction (XRD) analysis. Among the three, SC showed the strongest inhibitory effect on α-amylase and amyloglucosidase activities [24]. However, the precise mechanism behind this interaction remains to be fully elucidated.

This study employed fluorescence spectroscopy and fluorescence thermodynamic analysis to investigate the fluorescence quenching behavior, thermodynamic parameters, and quenching mechanisms of α-amylase and amyloglucosidase in the presence of NC, MC, and SC. Structural changes in both enzymes were also examined using synchronous fluorescence and UV–vis absorption spectroscopy. We hypothesized that decreasing crystallinity and increasing solubility of cellulose would enhance binding affinity and alter the microenvironment of key aromatic residues in α-amylase and amyloglucosidase. The findings proposed a strategy for preparing natural inhibitors targeting the activities of α-amylase and amyloglucosidase to effectively inhibit starch digestion, thereby providing theoretical support for the development of low-glycemic index (GI) foods and the adjuvant treatment of diabetes.

## 2. Materials and Methods

### 2.1. Materials

Corn seed coat (a mixed commercial batch) was obtained from COFCO Corporation (Beijing, China). α-amylase from porcine pancreas was purchased from Megazyme, and amyloglucosidase from Aspergillus niger (A7095, 300 units/mL) was obtained from Sigma-Aldrich (St. Louis, MO, USA). Sodium hydroxide, sodium chlorite, phosphoric acid (85%), calcium chloride, and other chemicals were supplied by Beijing Chemical Factory (Beijing, China). All reagents used were of analytical grade. Ultrapure water was produced using a Millipore Super Q system (Burlington, MA, USA).

### 2.2. Chemical Composition of Corn Seed Capsule

The chemical composition of corn seed coat, including starch (AOAC 2002.02), crude fat (AOAC 920.39), protein (AOAC 984.13), moisture (AOAC 934.01), ash (AOAC 942.05), total DF (AOAC 991.43), soluble DF (AOAC 991.43), and IDF (AOAC 991.43), was analyzed using Association of Official Analytical Chemists (AOAC) standard methods [25].

### 2.3. Extraction and Preparation of Cellulose from Corn Seed Capsule

Following the method described by Zhu et al. [24], the corn seed coat was washed, ground, and mixed with a chloroform–methanol solution (2:1, *v*/*v*) at a solid-to-liquid ratio of 1:20 (g/mL). The mixture was stirred at room temperature for 4 h, followed by centrifugation at 11,180× *g* for 20 min to collect the insoluble residues. These residues were dispersed in ultrapure water and adjusted to a pH of 6.0–6.6 with an acetate buffer consisting of acetic acid and sodium acetate trihydrate. Then, medium-temperature α-amylase (Shanghai Macklin Biochemical Technology Co., Ltd., China. A890336, 4000 U/g) was added, and the mixture was heated and stirred at 55 °C for 2 h. The starch-free residues were then treated with 2% sodium hydroxide solution at a solid-to-liquid ratio of 1:25 (*w*/*v*), heated at 80 °C for 2 h, and washed with ultrapure water until a neutral pH was reached. After drying, the residues were treated with 3% sodium chlorite solution containing 0.3% glacial acetic acid at 70 °C for 2 h (solid-to-liquid ratio of 1:30, *w*/*v*), and this process was performed once. The reaction was immediately stopped using an ice bath, and the material was washed with cold ultrapure water until neutral pH was achieved, followed by freeze-drying at −85 °C and 0.0014 mbar for 48 h to obtain white powdered NC.

For further processing, 10 g freeze-dried cellulose powder was added to 150 mL phosphoric acid (85%, *w*/*w*) and stirred until fully wetted. Thereafter, the cellulose dispersion in the beaker was placed into an ultrasonic cleaner (Model: KQ-100KDE; Manufacturer: Kunshan Ultrasonic Instrument Co., Ltd.; Location: Kunshan, China) for ultrasonic processing at 45 °C for 1 h, with the instrument operating at 45 W. Upon completion of acid hydrolysis, five times the volume of ethanol was added to terminate the reaction. The white precipitate was collected by filtration, washed repeatedly with ethanol until neutral, and dried. It was then redispersed in ultrapure water, stirred for 15 min, and centrifuged at 4000× *g* for 20 min. The precipitate (MC) and supernatant (SC) were collected separately and freeze-dried.

### 2.4. Interaction Assay Between Cellulose and Amylase

Suspensions of the three types of cellulose were prepared at various concentrations (0.003, 0.012, 0.021, 0.03 mg/mL) using sodium acetate buffer (0.4 M, pH 5.2 ± 0.1) and thoroughly mixed with α-amylase (30 U/mL) or amyloglucosidase (140 U/mL) at a ratio of 1:1 (*v*/*v*). The mixtures were reacted at 37 °C for 30 min. After centrifugation at 3000× *g* for 1 min, the interactions were characterized using fluorescence spectroscopy and UV–vis spectroscopy.

#### 2.4.1. Fluorescence Quenching Assay

Fluorescence parameters provide insights into alterations in the protein microenvironment. A fluorescence spectrophotometer (RF-6000, Shimadzu Corporation, Kyoto, Japan) was used to assess the fluorescence quenching of α-amylase and amyloglucosidase in response to cellulose addition. Parameter setting was performed using a published approach with some modifications [26]. Measurements were performed with an excitation wavelength of 285 nm, and emission spectra were collected from 290 to 440 nm at a scan rate of 6000 nm/min. Both excitation and emission slit widths were set to 5 nm. To determine the type of fluorescence quenching induced by cellulose on α-amylase and amyloglucosidase, emission spectra were recorded at three temperatures: 288 K, 298 K, and 308 K. Fluorescence spectra of cellulose alone under the same conditions served as the baseline for correction. To further elucidate the quenching mechanism and interaction type between cellulose and the enzymes, the fluorescence data were analyzed using the Stern–Volmer equation [27].

(1) $$\frac{F_{0}}{F}=1+K_{SV}[Q]=1+K_{q}\tau_{0}[Q]$$

where F_0_ is the fluorescence intensity of the protein before quencher addition, F is the fluorescence intensity of the protein after quencher addition, K_SV_ is the Stern–Volmer quenching constant, [Q] is the concentration of the quencher, Kq is the dynamic quenching constant, and τ_0_ is the fluorescence lifetime of biomolecules without quencher (typically τ_0_ = 10^−8^ s).

The modified Stern–Volmer equation was used to calculate the binding constant (K_A_) and binding site number (n) [28]:

(2) $$\log\left(\frac{F_{0}-F}{F}\right)=\log K_{A}+n\log[Q]$$

The thermodynamic parameters calculated by the van’t Hoff equation can determine the non-covalent force between protein and ligand [29]:

(3) $$\ln K=\frac{-\Delta H}{RT}+\frac{\Delta S}{R}$$

(4) ΔG=ΔH−TΔS

where K is the effective K_A_ at the corresponding temperature, T is the absolute temperature (K), R is the gas constant (8.314 J mol^−1^·K^−1^), ΔH is the enthalpy change, ΔS is the entropy change, and ΔG is the change in Gibbs free energy.

#### 2.4.2. Synchronous Fluorescence Spectroscopy

Synchronous fluorescence spectroscopy differs from conventional fluorescence spectroscopy by simultaneously scanning both the excitation and emission monochromators while maintaining a fixed wavelength interval. In this study, the wavelength intervals (Δλ) between excitation and emission were set at 30 nm and 60 nm. The slit widths were maintained at 5 nm, with a sampling interval of 1 nm and a scan rate of 6000 nm/min [30].

#### 2.4.3. UV–Vis Spectroscopy

Following the interaction between cellulose and either α-amylase or amyloglucosidase at 37°C, the samples were analyzed using a UV–Vis spectrophotometer (UV-2800A, Unico Instruments, Shanghai, China). Spectra were recorded over the wavelength range of 240–320 nm. Baseline correction was performed by subtracting the spectrum of the corresponding cellulose suspension at the same concentration was subtracted from the enzyme + cellulose spectrum [31].

### 2.5. Statistical Analysis

Statistical differences among the data (K_SV_, K_q_, K_A_) were analyzed using one-way ANOVA and a Duncan multiple comparison test at a significance level of 0.05. All experiments were conducted in triplicate to determine the mean ± standard deviation (SD). Initial data analysis was performed using Excel, and significance testing was performed with SPSS 19.

## 3. Results

### 3.1. Analysis of the Chemical Composition of Corn Seed Capsules

The chemical composition of corn seed capsules is presented in Table 1. The DF content of corn seed capsules is 76.4%, with IDF comprising 76.2% and soluble dietary fiber (SDF) making up only 0.2%. Apart from its high DF content, corn seed capsules also contain protein, starch, and trace amounts of fat and ash. Given that tens of millions of tons of byproducts are produced annually during corn processing, most of the corn bran is used as animal feed, while some is discarded as waste, leading to low-value addition and resource waste. Extracting cellulose from corn seed capsules could increase its use, create new markets for the deeper processing of corn bran, and increase its economic value.

### 3.2. Interaction Between Cellulose and Amylases

**Figure 1:**
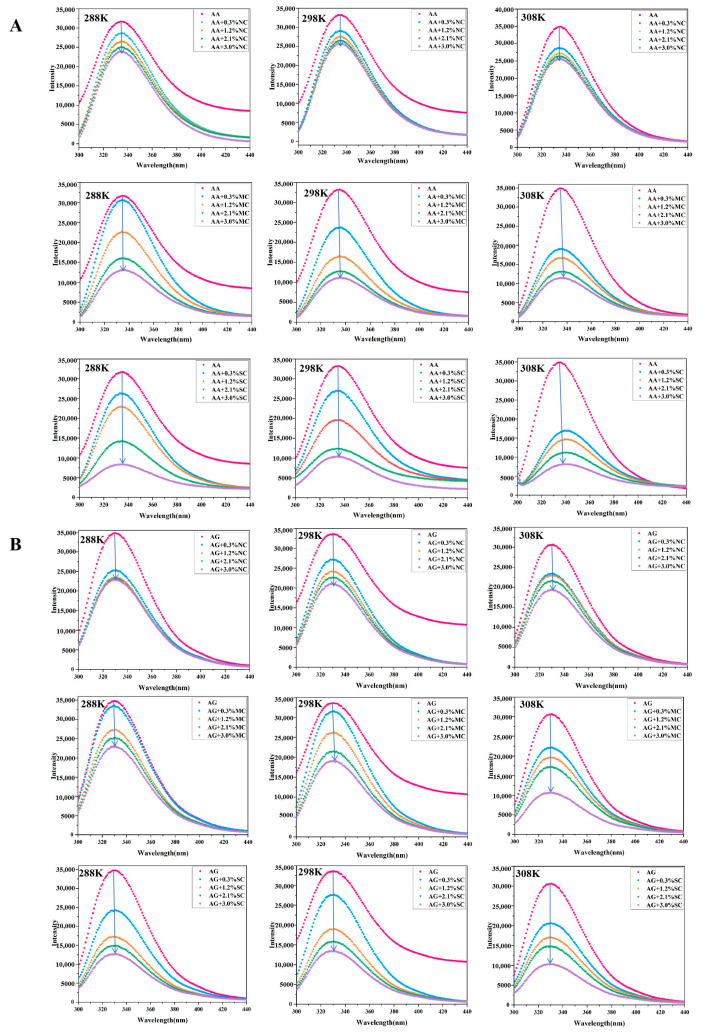
The fluorescence emission spectra with NC, MC, and SC (0%, 0.3%, 1.2%, 2.1%, 3.0%) on α-amylase (**A**) and amyloglucosidase (**B**) at 288 K, 298 K, and 308 K. Data are reported as the average measured value (n = 3).

Our previous research has provided preliminary insights into the effects of NC, MC, and SC on the activities of α-amylase and amyloglucosidase, and the results indicated that all three cellulose samples significantly reduced enzyme activity, with the inhibition increasing as the cellulose concentration rose [24]. NC, MC, and SC showed stronger amyloglucosidase inhibition than α-amylase [24]. Tryptophan, tyrosine, and phenylalanine are the inherent chromophores of α-amylase and amyloglucosidase. Studies have shown that the interaction between ligands and enzymes can be determined by measuring changes in fluorescence intensity and wavelength, thereby providing insights into conformational changes of the enzymes [32]. Therefore, the fluorescence spectra of a cellulose–amylase mixture at 288 K, 298 K, and 308 K were measured in this study. As shown in Figure 1, NC, MC, and SC caused fluorescence quenching in both α-amylase (A) and amyloglucosidase (B). This indicates that cellulose interacts with these enzymes to form complexes, altering the amino acid microenvironment and exposing the fluorescent groups to a polar environment, thereby reducing fluorescence intensity. In addition, as the sample concentration increased, the fluorescence intensity of α-amylase (Figure 1A) and amyloglucosidase (Figure 1B) gradually decreased, while the maximum peak wavelength of both enzymes exhibited a slight redshift. At 288 K, the addition of 3.0% (*w*/*v*) NC, MC, and SC reduced the maximum fluorescence intensity of α-amylase by 24.9%, 58.7%, and 73.7%, and that of amyloglucosidase by 34.2%, 34.3%, and 63.6%, respectively. This fluorescence quenching exhibited temperature dependence: further increasing the temperature to 308 K led to greater quenching efficiencies, with the maximum fluorescence intensity of α-amylase reduced by 26.8%, 67.2%, and 77.1%, and that of amyloglucosidase by 37.1%, 65.4%, and 66.4%, under the same additive concentrations. These results suggest that the addition of cellulose altered the enzyme conformation, leading to changes in the polarity of the tyrosine and tryptophan microenvironments and enhancing the hydrophilicity of the fluorescent group microenvironment. The difference in fluorescence intensity between different cellulose–enzyme mixtures may be attributed to the change in the interaction between the crystalline structure of cellulose and the active groups of α-amylase and amyloglucosidase.

#### 3.2.1. Fluorescence Quenching Mechanisms and Binding Affinities Analyzed by Stern–Volmer Plots

**Figure 2:**
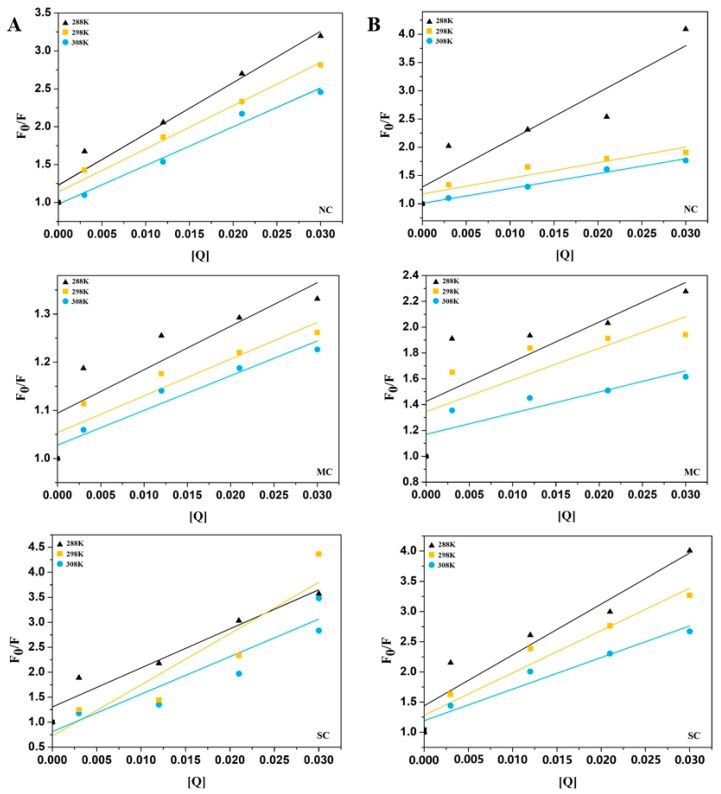
Stern–Volmer curves for the interaction of NC, MC, and SC with α-amylase (**A**) and amyloglucosidase (**B**) at 288 K (▲), 298 K (■), and 308 K (●).

Fluorophore quenching by a quenching agent is typically classified into dynamic quenching and static quenching. Dynamic quenching occurs when the quenching agent interacts with the excited fluorophore, while static quenching involves the interaction between the quenching agent and the ground-state fluorophore [27,33]. Figure 2 displays the Stern–Volmer curves for the interaction between amylases and celluloses with different molecular weights. The non-linear curves for NC, MC, and SC with both α-amylase and amyloglucosidase indicate that these cellulose samples quench fluorescence through static quenching mechanisms. In the present study, the Stern–Volmer quenching constants (K_SV_) were calculated and are presented in Table 2. For NC, MC, and SC, the K_SV_ values for α-amylase decreased with increasing temperature. According to fluorescence quenching theory, K_SV_ values increase with temperature for dynamic quenching. Therefore, it can be preliminarily concluded that the fluorescence quenching of α-amylase by these cellulose samples is predominantly due to static quenching.

At the same concentration, the quenching constants followed the order SC > MC > NC, suggesting that SC had the most significant fluorescence quenching effect on α-amylase. This may be attributed to changes in the cellulose crystalline structure and water solubility. A similar pattern was observed for the K_SV_ values of amyloglucosidase, consistent with previous fluorescence quenching findings. Unlike the fluorescence quenching parameters of α-amylase, for amyloglucosidase, the increase in K_SV_ values with rising temperatures and K_q_ values below the maximum collision quenching constant suggests that NC, MC, and SC induce dynamic quenching in amyloglucosidase. The K_A_ and binding site numbers (n) for α-amylase and amyloglucosidase at various temperatures are presented in Table 2. The K_A_ values for α-amylase interacting with NC, MC, and SC decreased as the temperature increased, supporting the static quenching mechanism inferred from the declining K_SV_. The K_A_ values indicate the strength of the binding between the quencher and the enzyme. The significantly higher K_A_ values for SC with both α-amylase and amyloglucosidase, compared to NC and MC, suggest that alterations in the crystalline structure of SC increase its affinity for the enzymes, in line with the fluorescence quenching observations.

#### 3.2.2. Thermodynamic Analysis Identifies Binding Forces Between Cellulose and Enzymes

The binding forces between the two biomolecules primarily involve hydrophobic interactions, hydrogen bonding, van der Waals forces, and electrostatic interactions. Based on the changes in ΔH and ΔS, the driving forces between the two biomolecules can be categorized as follows: (1) ΔH > 0 and ΔS > 0 indicate hydrophobic interaction as the main driving force; (2) ΔH < 0 and ΔS < 0 suggest van der Waals forces or hydrogen bonding as the primary driving force; (3) ΔH > 0 and ΔS < 0 point to electrostatic and hydrophobic interactions as the main forces; and (4) ΔH < 0 and ΔS > 0 indicate electrostatic forces as the predominant driving force. Based on the van’t Hoff equation, linear fitting was performed on the KA values measured at different temperatures. The correlation coefficient (R2) ranged from 0.991 to 0.998, indicating a good linear relationship, which confirms the reliability of the derived thermodynamic parameters (ΔH and ΔS). As presented in Table 3, the interaction between NC, MC, and SC with α-amylase showed that ΔH < 0 and ΔS < 0, implying that the binding was primarily driven by van der Waals forces or hydrogen bonding, with the process being exothermic. For amyloglucosidase, ΔH > 0 and ΔS < 0 suggest that hydrophobic interactions and van der Waals forces might be involved in the formation of the cellulose–amyloglucosidase complex, which was consistent with the study of Xing et al. [34]. The ΔG values of the mixtures of NC, MC, SC, and α -amylase are all negative, indicating that their binding to α-amylase is spontaneous. Furthermore, the larger the absolute value of ΔG, the greater the driving force of the spontaneous process. However, for amyloglucosidase, the ΔG values of NC, MC, and SC are positive, while the ΔS values are negative. This suggests that hydrophobic interactions and van der Waals forces may be involved in the formation of the cellulose–amyloglucosidase complex.

#### 3.2.3. Synchronous Fluorescence Spectroscopy Reveals Microenvironmental Changes in α-Amylase and Amyloglucosidase

Synchronous fluorescence spectroscopy scans both excitation and emission wavelengths simultaneously, maintaining a constant wavelength difference (Δλ) between them, which elevates sensitivity and reduces interference from scattered light. By setting Δλ = 30 nm and Δλ = 60 nm, this technique can detect changes in the microenvironment of tyrosine (Tyr) and tryptophan (Trp) residues [35].

**Figure 3:**
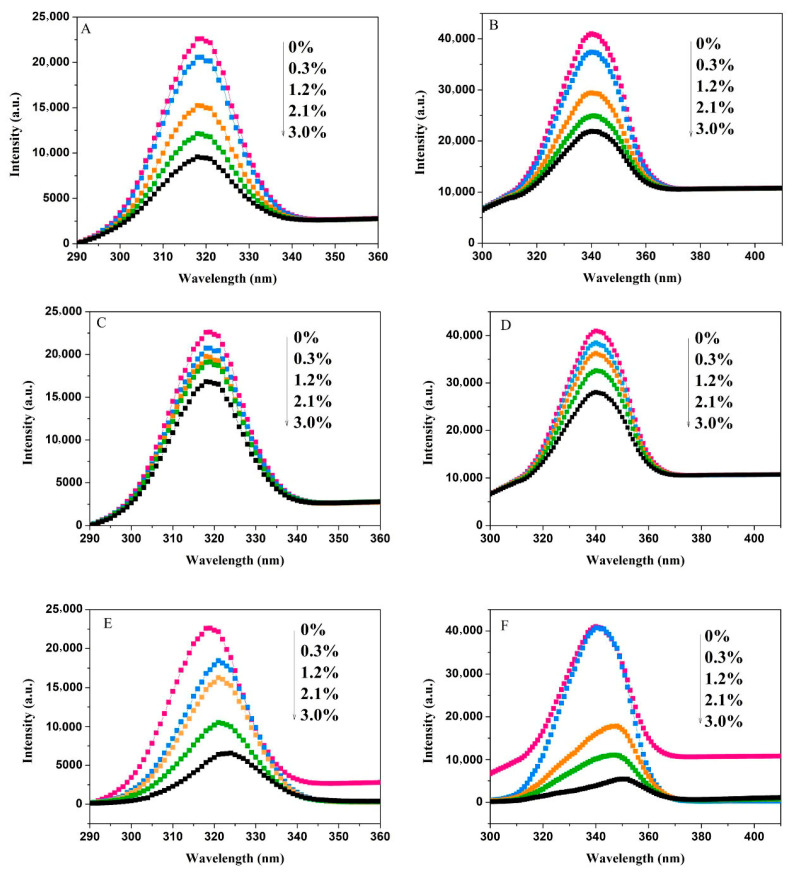
Synchronous fluorescence spectra of α-amylase interacting with NC (**A**,**B**), MC (**C**,**D**), and SC (**E**,**F**). (**A**,**C**,**E**) represent spectra obtained at Δλ = 30 nm, while (**B**,**D**,**F**) correspond to spectra with Δλ = 60 nm. (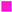: 0%, 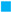: 0.3%, 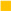: 1.2%, 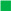: 2.1%, 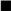: 3.0%).

As shown in Figure 3, the fluorescence intensity of Tyr and Trp residues in α-amylase decreased in the presence of NC (A, B), MC (C, D), and SC (E, F). The strength of fluorescence quenching increased with cellulose concentration. At a 3% cellulose concentration, the quenching degrees means of Tyr by NC, MC, and SC were 57.6%, 25.6%, and 70.9%, respectively, while the quenching degrees means of Trp were 46.6%, 31.6%, and 86.8%, respectively. These results suggest that changes in the cellulose crystalline structure affected its interaction with the enzyme, leading to varying impacts on the microenvironment of Tyr and Trp residues.

**Figure 4:**
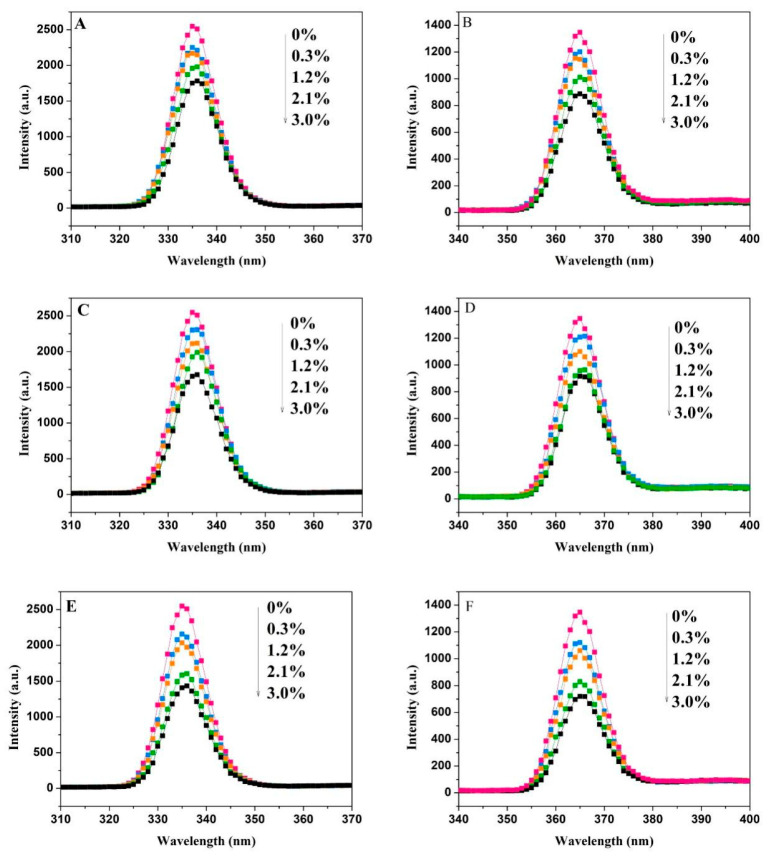
Synchronous fluorescence spectra of amyloglucosidase interacting with NC (**A**,**B**), MC (**C**,**D**), and SC (**E**,**F**). (**A**,**C**,**E**) represent the spectra at Δλ = 30 nm, while (**B**,**D**,**F**) correspond to spectra with Δλ = 60 nm. (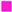: 0%, 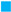: 0.3%, 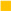: 1.2%, 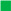: 2.1%, 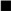: 3.0%).

As the concentration of SC increased, the maximum fluorescence emission peak of the tyrosine residue shifted from 319 nm to 324 nm, while the maximum fluorescence emission peak of the tryptophan residue shifted from 340 nm to 350 nm, both showing a redshift. This indicates that the hydrophobic interactions around the Tyr and Trp residues in α-amylase are weakened. Such a reduction in hydrophobicity facilitates the extension of the peptide chain, ultimately leading to conformational rearrangement of the enzyme. Figure 4 displays changes in the fluorescence intensities of Tyr and Trp residues in amyloglucosidase with varying concentrations of NC, MC, and SC. Fluorescence intensity decreased as cellulose concentration increased, accompanied by slight redshifts. SC has a disproportionately strong effect on Trp residues in both enzymes compared to NC and MC. At the highest concentration (3%), the quenching degrees of Tyr of NC, MC, and SC were 30.1%, 34.2%, and 43.7%, while for Trp, they were 10.7%, 32%, and 46.5% for NC, MC, and SC, respectively.

#### 3.2.4. UV–Visible Spectroscopy Indicates Static Quenching Mechanism

**Figure 5:**
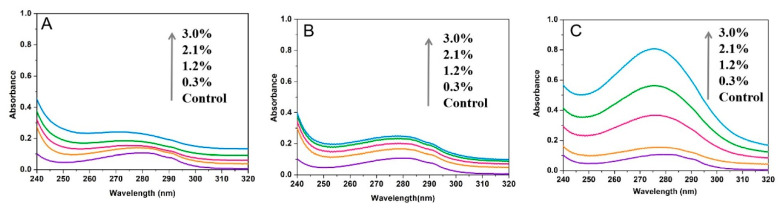
UV–visible absorption spectra of α-amylase in the presence of NC (**A**), MC (**B**), and SC (**C**). (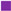: 0%, 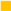: 0.3%, 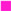: 1.2%, 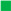: 2.1%, 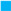: 3.0%).

**Figure 6:**
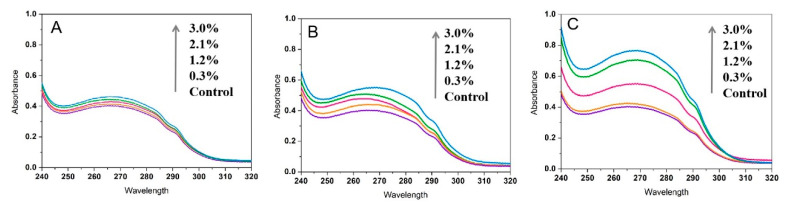
UV–visible absorption spectra of amyloglucosidase in the presence of NC (**A**), MC (**B**), and SC (**C**). ( 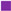: 0%, 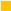: 0.3%, 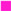: 1.2%, 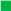:2.1%, 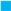: 3.0%).

UV–visible absorption spectroscopy serves as a complementary method for investigating fluorescence quenching mechanisms [31,36]. Figure 5 illustrates the UV absorption spectra of α-amylase after interaction with NC, MC, and SC. Compared to the control group, cellulose increased the absorption peak of α-amylase. With increasing cellulose concentration, the absorbance of α-amylase near 280 nm also increases, with SC showing the most significant effect. Based on this observation, we infer that the fluorescence quenching of α-amylase by NC, MC, and SC is static. These findings are consistent with the analysis of thermodynamic parameters. The addition of 3.0% (*w*/*v*) NC, MC, and SC increased the absorbance of α-amylase by 55.3%, 56.8%, and 86.6%, and that of amyloglucosidase by 12.7%, 26.8%, and 47.3% (Figure 6), respectively. Interestingly, the absorbance in the amyloglucosidase absorption spectrum also increases with increasing cellulose concentration, indicating the formation of ground-state complexes—that is, static quenching has occurred. However, this differs from the results of the thermodynamic analysis. The discrepancy may arise because as temperature increases, the enhancement of the dynamic quenching component “masks” the weakening of the static quenching component, leading to a thermodynamic manifestation where the Ksv value rises with temperature. Therefore, we conclude that NC, MC, and SC quench amyloglucosidase through a combination of dynamic and static quenching mechanisms.

## 4. Discussion

Reducing starch intake is the most effective way to prevent a sharp rise in blood glucose levels, though dietary habits based on starch as a staple food are challenging to change in the short term. While pharmaceutical treatments can lower blood glucose quickly, they often come with various side effects. As an alternative, researchers are exploring methods to inhibit digestive enzyme activity. For example, phenolic compounds can bind directly to digestive enzymes like amylase, sucrase, trypsin, and lipase, reducing their activity and slowing the digestion of starch and proteins [31]. Furthermore, the efficacy of plant-derived natural hypoglycemic substances, including polyphenol extracts (e.g., tea and coffee polyphenols [37]) and dietary fibers (e.g., pectin and coconut residue fibers [38,39]), has been extensively validated. As the primary component of dietary fiber, cellulose’s role in regulating enzyme activity is increasingly recognized, a fact that has generated significant research interest.

α-Amylase is a crucial digestive enzyme that breaks down starch into maltose and glucose, providing energy for the body. Amyloglucosidase is a biocatalyst that hydrolyzes the α-1,4 glycosidic bonds in starch, soluble starch, and related oligosaccharides [40]. Research by Nsor-Atindana et al. [16] showed that reducing the particle size of nanocrystalline cellulose increased its effect on amylase activity, inhibiting starch hydrolysis and slowing the rapid rise in glucose levels. In the present study, we demonstrated that celluloses with varying crystalline structures inhibited the activity of both α-amylase and amyloglucosidase through different mechanisms, with SC showing the most significant fluorescence quenching effect. The main reason for this phenomenon is that the amorphous structure of SC enhances its affinity for enzymes, promoting SC to bind to α-amylase via hydrogen bonds and van der Waals forces, reducing the hydrophobicity of the α-amylase microenvironment, and ultimately lowering the enzyme activity. This point is well supported by thermodynamic analyses and the redshift of synchronous fluorescence spectra.

The reduced effect of MC on α-amylase and amyloglucosidase can be attributed to the acid hydrolysis process, which alters the amorphous structure of cellulose and increases its crystallinity. The MC particles formed a bundle-like aggregated structure. Despite an increase in specific surface area, there were fewer accessible hydroxyl groups and reduced surface roughness, thus weakening the interactions between MC and the enzymes compared to the amorphous SC sample. The interactions between NC, MC, SC, and amyloglucosidase were mainly driven by hydrophobic interactions, which inhibited amyloglucosidase activity. In line with previous findings from the present research, acid hydrolysis disrupted the crystalline cellulose structure, resulting in loose, short-chain structures with larger specific surface areas and greater exposure to binding sites for enzyme interactions. Naturally, the conditions employed in this study have certain limitations—for instance, in vitro conditions (buffer, enzyme concentrations) may not represent physiological conditions. These limitations should therefore be validated through more rigorous experimental designs.

## 5. Conclusions

This study focused on the inhibitory effects and mechanisms of NC, MC, and SC on α-amylase and amyloglucosidase. The results showed that SC exhibits the highest binding affinity and strongest quenching effects. The crystalline structure of cellulose plays a key role in determining the strength of these interactions. NC, MC, and SC primarily bind to α-amylase through van der Waals forces or hydrogen bonding, while hydrophobic interactions dominate their binding with amyloglucosidase. These interactions reduce the hydrophobicity around Tyr and Trp residues, alter enzyme conformation and activity, and ultimately inhibit starch digestion [24]. In future research, it is necessary to focus on analyzing the regulatory effects of cellulose on the texture and sensory attributes of starch-based foods during low-GI product development, while integrating in vitro and in vivo starch digestibility assessments (e.g., glycemic glucose release kinetics, postprandial glycemic response) to systematically clarify the hypoglycemic mechanism of cellulose and its practical applicability in functional food formulation.

## Figures and Tables

**Table 1:** Elementary composition of corn seed capsule (g/100 g, dry weight basis).

Component	Protein	Moisture	Starch	Fat	Ash	DF	IDF	SDF
Corn seed capsule	6.9 ± 0.3	3.1 ± 0.1	2.9 ± 0.3	0.9 ± 0.1	0.8 ± 0.1	76.4 ± 0.5	76.2 ± 0.1	0.2 ± 0.1

**Table 2:** Stern–Volmer quenching constants (K_SV_) and binding constants (K_A_) for NC, MC, and SC interacting with α-amylase and amyloglucosidase at 288 K, 298 K, and 308 K.

	Samples	T (K)	K_sv_ (L/mol)	K_q_ (×10^9^ L·mol^−1^·s^−1^)	K_A_ (L·mol^−1^)	n
α-amylase	NC	288	6.078 ± 0.125 ^e^	0.061 ± 0.125 ^e^	1.781 ± 0.016 ^d^	0.581 ± 0.022
		298	5.419 ± 0.212 ^e^	0.054 ± 0.212 ^e^	1.324 ± 0.014 ^d^	0.355 ± 0.020
		308	5.227 ± 0.036 ^e^	0.052 ± 0.036 ^e^	1.147 ± 0.079 ^d^	0.242 ± 0.016
	MC	288	66.107 ± 1.301 ^c^	6.610 ± 1.301 ^c^	29.747 ± 0.118 ^c^	1.305 ± 0.062
		298	66.043 ± 1.018 ^c^	6.604 ± 1.018 ^c^	25.975 ± 2.197 ^c^	0.710 ± 0.030
		308	29.508 ± 1.594 ^d^	2.951 ± 1.594 ^d^	4.073 ± 0.280 ^d^	0.319 ± 0.007
	SC	288	114.293 ± 9.084 ^a^	11.429 ± 9.084 ^a^	84.705 ± 1.259 ^a^	1.047 ± 0.011
		298	83.478 ± 7.023 ^b^	8.348 ± 7.023 ^b^	55.305 ± 1.982 ^b^	1.012 ± 0.011
		308	65.718 ± 3.965 ^c^	6.572 ± 3.965 ^c^	11.262 ± 1.405 ^d^	0.452 ± 0.001
amyloglucosidase	NC	288	9.386 ± 0.246 ^h^	0.936 ± 0.246 ^h^	1.263 ± 0.028 ^g^	0.222 ± 0.005
		298	10.496 ± 0.483 ^h^	1.050 ± 0.483 ^h^	1.707 ± 0.046 ^g^	0.164 ± 0.007
		308	13.289 ± 0.033 ^g^	1.329 ± 0.033 ^g^	1.746 ± 0.092 ^g^	0.120 ± 0.010
	MC	288	20.599 ± 0.804 ^f^	2.060 ± 0.804 ^f^	4.211 ± 0.413 ^f^	0.429 ± 0.016
		298	33.386 ± 0.138 ^e^	3.339 ± 0.138 ^e^	6.686 ± 0.038 ^e^	0.480 ± 0.007
		308	54.956 ± 0.010 ^c^	5.496 ± 0.010 ^c^	8.215 ± 0.284 ^d^	0.486 ± 0.005
	SC	288	44.309 ± 0.258 ^d^	4.431 ± 0.258 ^d^	12.315 ± 0.858 ^b^	0.572 ± 0.021
		298	58.923 ± 0.126 ^b^	5.892 ± 0.126 ^b^	15.257 ± 0.155 ^a^	0.549 ± 0.002
		308	76.791 ± 0.513 ^a^	7.679 ± 0.513 ^a^	11.168 ± 0.227 ^c^	0.407 ± 0.006

Different letters in the same column within each enzyme indicate significant differences among groups (*p* < 0.05).

**Table 3:** Thermodynamic parameters for interacting NC, MC, and SC with α-amylase and amyloglucosidase at various temperatures.

	Samples		ΔH (kJ·mol^−1^)	ΔS (J·mol^−1^·K^−1^)	ΔG (kJ·mol^−1^)
α-amylase	NC	288 K	−21.17 ^c^	−1.37 ^b^	−68.23 ^a^
		298 K		−0.69 ^a^	
		308 K		−0.33 ^a^	
	MC	288 K	−49.94 ^a^	−8.12 ^e^	−145.07 ^c^
		298 K		−8.06 ^e^	
		308 K		−3.61 ^c^	
	SC	288 K	−30.42 ^b^	−10.60 ^g^	−71.88 ^b^
		298 K		−9.93 ^f^	
		308 K		−6.22 ^d^	
amyloglucosidase	NC	288 K	21.50 ^b^	−0.56 ^a^	75.70 ^b^
		298 K		−1.32 ^b^	
		308 K		−1.43 ^b^	
	MC	288 K	32.99 ^a^	−3.43 ^c^	125.62 ^a^
		298 K		−4.71 ^d^	
		308 K		−5.43 ^e^	
	SC	288 K	15.29 ^c^	−6.01 ^f^	72.42 ^b^
		298 K		−6.75 ^g^	
		308 K		−6.22 ^f^	

Different letters in the same column within each enzyme indicate significant differences among groups (*p* < 0.05).

## Data Availability

The original contributions presented in this study are included in the article. Further inquiries can be directed to the corresponding author.
